# Teaching Tricks of the Trade: Shaking Up Gerontology Curriculum Through Pedagogical Innovation

**DOI:** 10.1093/geroni/igaf122.1586

**Published:** 2025-12-31

**Authors:** L B Bouchard, Rona Karasik

## Abstract

The Academy for Higher Education in Gerontology (AGHE) provides comprehensive competencies for gerontology education that can guide pedagogical innovations in gerontology curriculum. With these guidelines in mind, this symposium will showcase fresh activities, strategies, and partnerships that promote meaningful student engagement and learning outcomes. Speaker one will discuss a community based learning program that trains students to gain beneficial volunteer experience as resident advocates for older adults in long term care. Speaker two will discuss a course project where students created podcast episodes about familial relationships based on interviews they conducted with older adults. Speaker three will discuss an ungrading approach that promotes detailed, qualitative feedback and reflection instead of traditional numerical grading. Speaker four will discuss a rich departmental partnership to prepare students for practicum utilizing poetic portraiture and application of virtual reality tools. Speaker five will discuss a role playing game to promote deeper understanding of cumulative inequality among undergraduate gerontology students.

